# Editorial: Recent advances in anti-doping

**DOI:** 10.3389/fspor.2025.1636184

**Published:** 2025-06-19

**Authors:** Raphael Faiss, James Hopker

**Affiliations:** ^1^ REDS, Research & Expertise in AntiDoping Sciences, Institute of Sport Sciences, University of Lausanne, Lausanne, Switzerland; ^2^ School of Sport, Exercise & Rehabilitation Sciences, University of Kent, Canterbury, UK

**Keywords:** doping, anti-doping, detection, education, deterrence, integrity

**Editorial on the Research Topic**
Recent advances in anti-doping

The fifth revision of the World Anti-Doping Code will come into effect in 2027, after thorough amendments made in the 2021 version come into effect (1). The evolving regulatory framework has significantly influenced research in anti-doping science and integrity in the past years, particularly with the revised definition of substances of abuse, the concept of aggravating circumstances, and the broader inclusion of policies regarding potential unintentional contamination cases. This research topic (RT) provided an opportunity for researchers to share new insights and novel developments as well as future perspectives in the field of Anti-Doping Sciences. Studies focused on optimizing adherence to legal principles by supporting education, detection and prevention of both deliberate and inadvertent doping.

Pelobello et al. identified medical practitioners as well positioned to prevent doping among athletes as they are a trusted resource for their (athlete) patients. They evaluated an education program in medical students to underscore the importance of iterative curriculum development in medical education, particularly when introducing novel topics like drugs in sport. In parallel, a study on amateur gym-goers outlined the need for targeted interventions to address misconceptions and promote safer practices, particularly for use of nutritional supplements potentially leading to an abuse of anabolic steroids (AlKasasbeh et al.). Education was also highlighted as central for law enforcement authorities to allow intensified prevention efforts at gyms (Kvillemo et al.). However, amateur athletes do not necessarily have a lenient attitude towards doping, but over-the counter use of medication may certainly increase the risk of unintentional doping (de Abreu et al.). The well-established use of supplements was also reported in elite athletes. Myoenzono et al. identified that a large proportion of Olympians and Paralympians (from 50%–70%) used supplements, with unregulated use presenting high risks, like overdosing and/or anti-doping rule violations. Such results also echoed in the perceptions of anti-doping policy and practice of the anti-doping system by elite Para-athletes (Qvarfordt et al.) with unique conditions faced by athletes with impairments within the anti-doping system.

Nevertheless, initiatives to recenter the athletes (or para-athletes) at the core of the anti-doping activities also emerged from the 2021 WADA Code. For instance, categorization of the situations of vulnerability that converge toward doping in sport were proposed (Filleul et al.). In that study, four types of vulnerability situations were outlined: (i) psychological, (ii) physical (iii) relational, and (iv) contextual. Schneider et al. advocated in turn for an “urgent implementation of comprehensive safeguarding measures that address the vulnerabilities associated with anti-doping amongst athletes at all levels”.

The detection and enforcement of anti-doping rules require robust testing programs. In that context, loopholes in sports with growing interest from participants and media illustrate specific challenges for anti-doping stakeholders to succeed in implementing robust policies. For example, financial constraints, infrastructural and logistical barriers, and cultural factors were identified as challenges for enforcing anti-doping measures in ultramarathon (Colangelo et al.). Beyond contextual challenges for anti-doping policymakers, significant scientific advances have been made in testing methods over the past decade. For example, Oliveira et al. presented new biomarkers to tackle blood doping with a routine application possibly implemented without significant logistical or analytical constraints. In addition, as shown by analysis of performance data from female weightlifters (Ryoo et al.), advanced artificial intelligence algorithms may leverage more efficient and objectively targeted anti-doping tests in the near future.

In conclusion, this Research Topic aimed to deliver expert-driven, well-documented insights to address contemporary challenges in anti-doping efforts. The collected body of evidence reinforces evidence-based approaches for stakeholders by better defining priorities and strategies to deter athlete misconduct. The included studies spanned novel original research offering pragmatic perspective to questions emerging from the daily enforcement of the Code. Ultimately, this Research Topic facilitated the consolidation and discussion of existing evidence, presenting practical proposals and empirical findings to fortify the fight against doping.
